# Evaluation of adipose tissue changes with bioimpedance and dual-energy X-ray absorptiometry in treatment of multi-experienced HIV-infected patients

**DOI:** 10.1186/1471-2334-14-S4-P3

**Published:** 2014-05-29

**Authors:** Raluca Mihăilescu, Daniela Ioana Munteanu, Mihai Lazăr, Cătălin Tilişcan, Laura Papagheorghe, Raluca Dulama, Loredana Benea, Mihaela Rădulescu, Ana Maria Tudor, Adrian Streinu-Cercel, Adriana Hristea, Anca Ruxandra Negru, Irina Lăpădat, Daniela Ion, Sorin Ștefan Aramă, Victoria Aramă, Adrian Streinu-Cercel

**Affiliations:** 1 National Institute for Infectious Diseases “Prof. Dr. Matei Balş”, Bucharest, Romania; 2 Carol Davila University of Medicine and Pharmacy, Bucharest, Romania

## 

Multi-experienced HIV-infected patients bear the burden of treatment-related toxicities over the years. This study evaluated the correlation between bioimpedance- and DXA-quantified changes of adipose tissue with duration of infection and duration of combined antiretroviral therapy (cART) in HIV-seropositive patients.

A cross-sectional study, belonging to prospective grant PNCDI2 no.62077/2008, was conducted in a national reference hospital in 2011-2012. DXA whole-body adipose tissue analysis and bioimpedance analysis revealed fat and lean tissue (% and grams), android/gynoid distribution, arm+legs/trunk ratio and waist/hip ratio (WHR).

There were 78 patients enrolled, including the control seronegative group. HIV-seropositive patients had equal sex distribution, median age of 33 years with mode of 20 years and body mass index of 23.6 kg/sqm [21;25.7]. Duration of diagnosed infection, duration of cART and number of previous therapeutic regimens had medians of 69 months [36;113], 57 months [27;111] and 2 [1;3], respectively. Among all variables, WHR correlated with duration of infection (rho= -0.36, p=0.05), duration of cART (rho= -0.36, p=0.05) and duration of number of therapeutic regimens (rho= -0.48, p=0,007). Also fat and lean tissue, as grams, correlated with duration of cART - rho= -0.26, p=0.048 for both.

In this young treatment-experienced HIV-infected population undergoing antiretroviral therapy, the waist/hip ratio correlated best with the number of previous therapeutic regimens, inversely proportional. Adipose changes, measured by bioimpedance and DXA, were rather correlated with treatment duration than with time from diagnosis. Patients experiencing the history of antiretrovirals still present adverse effects on long term, which could influence their adherence to antiretrovirals.

